# Chewing efficiency in children with motor speech disorders

**DOI:** 10.1007/s40368-025-01095-6

**Published:** 2025-08-20

**Authors:** H. Björelius, G. Tsilingaridis, F. Johansson, J. Trang, A. Grigoriadis, R. Thorman, H. Terband

**Affiliations:** 1 Karolinska Institutet, Department of Clinical Sciences, Danderyd Hospital, Stockholm, Sweden; 2 Centre for Eating, Speech and Oral Motor Function, Division of Speech and Language Pathology, Department of Neurology, Danderyd Hospital, Stockholm, Sweden; 3 Karolinska Institutet, Department of Dental Medicine, Division of paediatric dentistry, Stockholm, Sweden; 4 Medical library, Danderyd Hospital, Stockholm, Sweden; 5 Center of Paediatric Oral Health Research, Stockholm, Sweden; 6 Karolinska Institutet, Department of Dental Medicine, Division of Oral Rehabilitation, Stockholm, Sweden; 7 Department of Communication Sciences and Disorders, University of Iowa, Iowa City, IA USA

**Keywords:** Chewing efficiency, Motor speech disorders, Speech sound disorders, Quality of life

## Abstract

**Purpose:**

This study aimed to investigate chewing efficiency in children with speech sound disorders (SSD). With a focus on those with motor speech disorders (MSD).

**Method:**

A clinical sample of 101 children with SSDs (78 with MSD), aged 4–9 years, and a control group of 76 typically developing (TD) children participated. Chewing efficiency was assessed using the two-colour Hue-Check^©^ chewing gum test. A computer-based image analysis measured colour mixing after 20 chewing cycles, generating the outcome variable SDHue.

**Results:**

An independent samples t-test showed that children with SSD chewed less efficiently than TD children. A one-way ANOVA revealed that children with MSD + (i.e., all children with MSD who also had concomitant language-oriented diagnoses [LD] and/or oral motor developmental delay [ODD]) aged 7–9 years had significantly lower chewing efficiency than age-matched TD peers (*p* < 0.001, *η*^2^ = 0.305).

**Conclusion:**

Children with MSD demonstrated reduced chewing efficiency compared to their TD peers. Possibly reflecting a broader, not yet fully understood, symptom complex. Oral motor developmental delay (ODD) was common across the entire SSD group. These findings underline the importance of future research exploring symptom interrelations and guiding targeted interventions.

**Supplementary Information:**

The online version contains supplementary material available at 10.1007/s40368-025-01095-6.

## Introduction

Evidence regarding chewing efficiency in children with speech sound disorders (SSD) is limited. Especially in children under the age of 6 years and diagnosed with a motor speech disorder (MSD) (Mogren et al. 2020). The ability to chew efficiently is fundamental. Complications may present themselves as refusal of food, being picky eaters, consuming too much food, or swallowing food without chewing (Morris 2000). Symptoms of inefficient chewing can be associated with delays in oral-motor and oral-sensory functions. Both in children with unknown causes (McAllister and Lundeborg 2013), and in those with diagnosed neurodevelopmental conditions (Maynard et al. 2020). Inefficient chewing has also been linked to functional difficulties, as well as negative impacts on emotional and social well-being (Abd-Elsabour et al. 2022; Morris 2000).

### Speech sound disorders

Paediatric SSDs have been found to be one of the most prevalent developmental disorders among children (Bishop 2010). SSDs comprise a wide range of underlying origins and causes. Such as language- motor, anatomical, and/or structural origins (ASHA 2025; Maassen and Terband 2024; Namasivayam et al. 2020; Shriberg et al. 2019). The prevalence of SSD is between 3 and 4 per cent of the population (Eadie et al. 2015). Motor speech disorders (MSDs) are diagnoses under the umbrella term SSD that can have a genetic, acquired, or idiopathic origin (Morgan et al. 2024; Shriberg et al. 2019). About 3 to 12 per cent of the children with idiopathic SSDs have been found to have an MSD diagnosis (Shriberg et al. 2019). The symptoms in MSD lead to orofacial and speech difficulties, that affect the control, coordination, and planning of speech movements. Resulting in unintelligible speech production and severe disruption in communication. The differentiation between MSD subtypes is not entirely solved due to the complexity of overlapping symptoms (ASHA 2007, 2025; Iuzzini-Seigel et al. 2022; Maassen and Terband 2024; McCabe et al. 2024; Murray et al. 2021). The primary subtypes of MSDs are Childhood apraxia of speech (CAS), Dysarthria (DYS), Oral dyspraxia (OA), and Speech motor delay (ATYP) (Shriberg et al. 2019). Childhood apraxia of speech is a neurological, sensorimotor speech disorder characterised by problems in motor planning and programming of the movements needed for speech (ASHA 2007). Dysarthria is a speech disorder of the neuromuscular execution of muscle movements involved in speech (Iuzzini-Seigel et al. 2022; Simmons and Mayo 1997). Oral dyspraxia impacts the volitional control of orofacial muscles in non-speech oral gestures and can cause prominent problems with speech development (Bearzotti et al. 2007; Hayden 1994). Speech motor delay has been identified as the most common subtype of MSD and is believed to be a part of an idiopathic neurodevelopmental delay (Shriberg et al. 2019). Speech motor delay should be considered when difficulties are seen in speech motor control and coordination of speech sounds, but after ruling out CAS/OA and/or DYS (Namasivayam et al. 2023). In addition to impaired speech, children with MSD often present with a range of co-occurring vulnerabilities. One such condition is oral motor developmental delay (ODD). Although not classified as a speech diagnosis, it may impact speech development and production due to delayed acquisition of oral motor milestones (Hayden and Square 1999; Kent 2015, 2021). ODD encompasses persistent oral-motor and oral-sensory functions and habits that extend beyond what is typically expected in development (Bakke et al. 2007; Murray et al. 2023). Furthermore, children with MSD may exhibit difficulties in sensory processing (Kent 2024; Newmeyer et al. 2009), fine and gross motor coordination (Tükel et al. 2015) and language development (Murray et al. 2021). Making such children a highly heterogeneous population.

### Mastication, oral- and speech motor development

Although the process of chewing might seem easy, it is a complex process consisting of concomitant neurological, physiological, motor, and sensory activity under strict regulation by central pattern generators allocated to the brainstem (Barlow et al. 2010). More specifically, muscular movements of the jaw are governed by a coordinative structure including the masseter, medial, and lateral pterygoid and temporalis muscles, innervated by the mandibular branch of the trigeminal nerve (V). Precise control of movement (controlling strength, speed, and fine-tuning displacement trajectories) relies on the function of the proprioceptive receptors of the muscle spindles, periodontium, (Almotairy et al. 2020) and Golgi tendon guard in the temporal mandibular joint (TMJ) (Frayne et al. 2016; Van der Glas et al. 2007). Chewing movements are initiated by the central pattern generators. Modulated by inputs from the peripheral orofacial receptors and inputs from the higher centres of the brain (Grillner 1982). Mandibular control and the coordinative and organised infrastructure needed for oral motor skills such as sucking, chewing, and babbling are found to be very similar in toddlers as young as 9 months old compared to older toddlers and adults (Steeve et al. 2008). Maturation of oral motor behaviours seems to develop sequentially over time and relies on the capacity to use the muscles involved in oral motor tasks (Cheng et al. 2007; Green et al. 2002; Steeve et al. 2008). Maturation reaches its high point at the age of 6 years and then levels out (Bearzotti et al. 2007). It has been shown that the sensorimotor mechanism governing the oral functions, such as biting and chewing, demonstrate a transition to the “adult-type” of behaviour at an age of 10 to 14 years (Almotairy et al. 2021, 2018, 2020). Furthermore, in healthy adults, the speech motor commands and non-speech oral motor movements, which both share the same muscular and neurological network structure (Kent 2015), are not linked and serve from different underlying patterns (Bunton 2008; Weismer 2006). Studies on children with persistent SSD after the age of 6 years, found asymmetrical bilateral movements of the lips and jaw when producing vowels. Compared to children with no speech dysfunctions (Mogren et al. 2022a, b). This could indicate that children with SSD have less stability in the lips and the TMJ that is needed for smooth speech motor commands as well as in chewing behaviours.

### Chewing efficiency in children

The ability to chew efficiently is fundamental, and complications may present themselves as refusal of food, being picky eaters, consuming too much food, or swallowing food without chewing (Morris 2000). Chewing skills among typically developing children (TD) have been investigated to some extent, and individual differences in chewing efficiency have been found to be substantial (Almotairy et al. 2021; Alshammari et al. 2022; Kaya et al. 2017; Mogren et al. 2022a, b). Oral handling of different types of food is a skill found in children as young as six months of age, though not fully developed (Gisel 1991). By the age of two, TD children can chew and process many types of food textures (Gisel 1991; Green et al. 1997). A recent study found that TD children as young as 3 years of age, have jaw motor functions needed for chewing similar to adults (Almotairy et al. 2021). As children grow older, chewing behaviour changes when the chewing duration before swallowing decreases, but not the number of chewing cycles. The number of cycles and their duration are dependent on food hardness, and are under development from childhood to adolescence, becoming adult-like first at the age of 15 years (Almotairy et al. 2021; Gisel 1991). Kaya et al. (2017) compared chewing efficiency in 25 children (mean age 10 years) to 27 adults and found that children chewed less efficiently than adults.

Malocclusion in children is common and the impact of it on chewing efficiency has been studied to some extent. A systematic review by Alshammari et al. (2022), found that malocclusion in the primary dentition in children aged 3–5 years, affects the chewing efficiency negatively. Compared to age matched controls without malocclusion. Furthermore, Mogren et al. (2022a, b) investigated the effect of malocclusion on chewing efficiency in a sample of 6- to 18-year-old children. It included both children with TD and children with SSD and found no differences between or within the groups (Mogren et al. 2022a, b).

In summary, chewing skills appear to develop progressively with age in children with TD (Almotairy et al. 2021; Gisel 1991). Although chewing efficiency is known to impact quality of life (Abd-Elsabour et al. 2022; Chen & Engelen 2012), studies in clinical populations remain scarce. There is a lack of research focusing on children with MSD, despite their known high rate of oral-motor difficulties. This highlights a clear need for further investigation of chewing skills in this group.

### Aim of the present study

The aim of the present study is to investigate chewing efficiency in a clinical population of children diagnosed with SSD, and specifically children with an MSD diagnosis, and compare the outcomes with TD children.

## Method and procedures

### Participants

The participants were recruited from the Centre for Eating, Speech and Oral Motor Function (OMC) at the Division of Speech and Language Pathology, in Stockholm, Sweden, which handles approximately 500 referrals per year regarding speech motor and oral motor disorders. The participants in the present study were drawn from a consecutive sample of children referred to the OMC between January 2022 and October 2023 as part of a larger research project including 211 children. Five speech-language pathologists were involved in collecting clinical samples, and informed consent was provided by the child’s caregiver when visiting the clinic. To meet the criteria for participation, children had to be between four and nine years of age, have one caregiver who could speak and read Swedish, agree to participate in the chewing task, and be diagnosed with an SSD. Children in these groups were not included; those with solely an oral motor developmental delay diagnosis (ODD) (*n* = 14), a structural diagnosis (ankyloglossia, hypertrophy of tonsils and/or adenoid), or not having received any diagnosis (*n* = 7). The final number of children with SSD in the present study was 101 (69 boys and 32 girls). Of the 101 children with SSD, 78 had MSD and 23 LD (Table 1). Data from an age matched control group (CG) of 77 (37 boys and 40 girls) TD children were collected between September 2022 and March 2024 in a consecutive manner during their regular dental examinations at; the Division of Paediatric Dentistry at Karolinska Institute, at a private dental clinic, and from colleagues’ children at the Division of Speech and Language Pathology. To be included in the control group, one parent had to be able to speak and read Swedish, the child should not have received any intervention from an SLP, and they should not have any known general developmental delay or dysfunction. Of the 77 children, 76 (36 boys and 40 girls) agreed to participate in the chewing task (CG). Caregivers of all included children provided informed consent.

**Table 1:** Sample of entire study group

Descriptives of studied sample *N* = 177				
Age 4 to 9 years:months	CG (*n* = 76)		SSD (*n* = 101)	
Gender	Boy	Girl	Boy	Girl
SSD total	*n*=36	*n*=40	*n*=69	*n*=32
Mean age	(6:1)	(6:8)	(6:2)	(6:2)
Min max age	4:0–9:2	4:0–9:4	4:2–9:9	4:5–9:4
MSD +				
Total			*n* = 52	*n* = 26
Mean age			(6:5)	(6:5)
LD +				
Total			*n *= 17	*n* = 6
Mean age			(6:0)	(5:9)

**Fig. 1:**
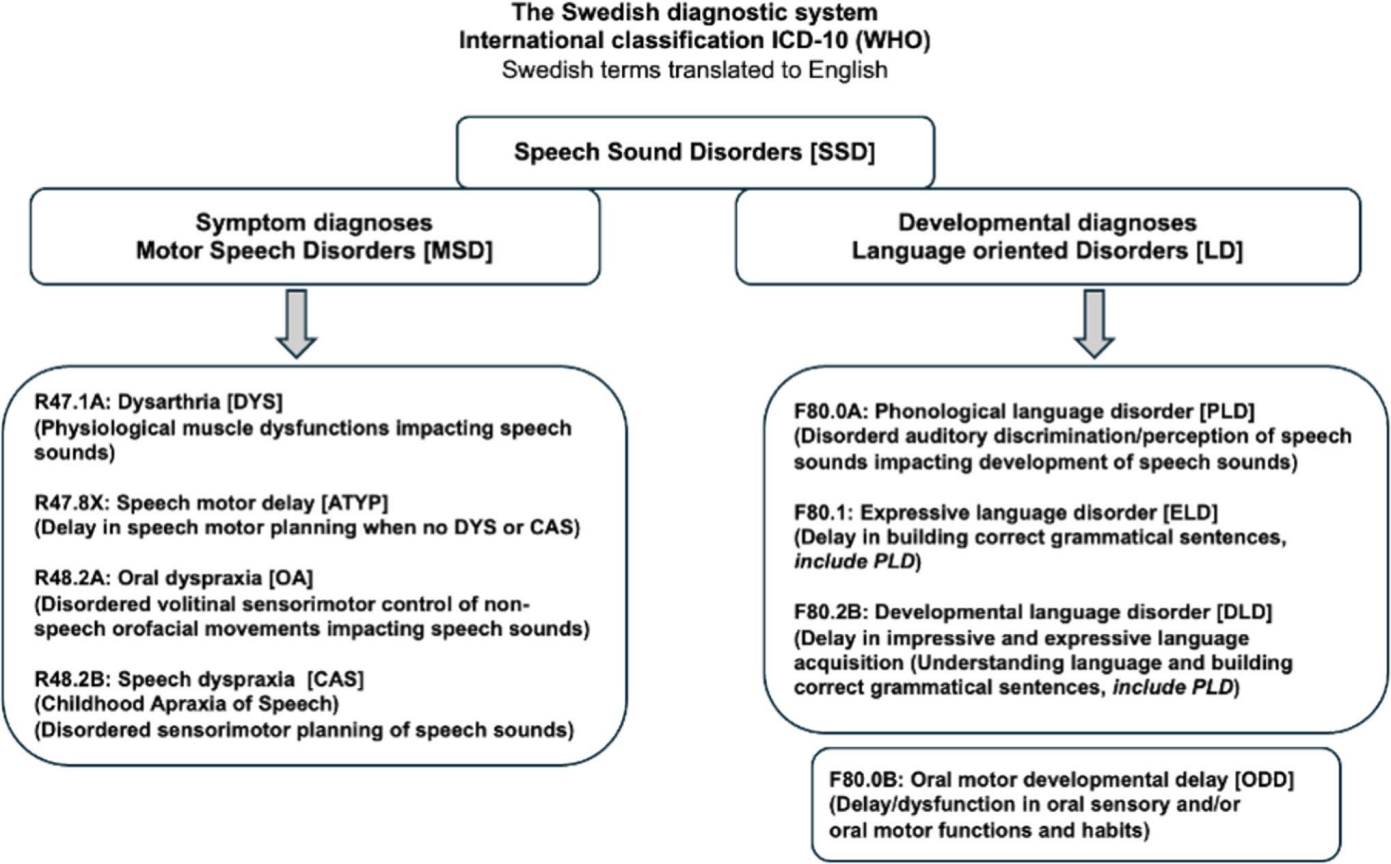
Flowchart describing the diagnoses from ICD-10 (WHO) used in clinical settings by speech language pathologists (SLPs) for children and adults in Sweden. Diagnostic terms translated from the Swedish to English

Gender, mean, minimum and maximum age presented. See Fig. 1 for detailed description of specific MSD and LD diagnoses

*CG* control group, *SSD* speech sound disorders, *MSD* + motor speech disorders, *LD* + language-oriented speech disorders

### Diagnostics and terminology

Speech Language Pathologists in Sweden use diagnostic terms, codes, and criteria based on ICD-10 (WHO 2004), which do not always correspond directly to terms used in the English SLP literature. To enable comparison, the OMC team selected the closest equivalent English terms based on the original Swedish ICD-10 diagnoses (Fig. 1). Differential diagnostics at OMC incorporates assessment of potential MSDs, phonological language disorder (PLD) and ODD (Appendix 1a). In Sweden, the criteria for diagnosing PLD include dysfunctions in the auditory discrimination and perception of speech sounds affecting the production of specific speech sounds. Language impairment is not assessed at OMC, but some children had been diagnosed with a specific language impairment, i.e. developmental language disorder (DLD) or expressive language disorder (ELD), prior to the assessment at OMC. All language oriented diagnoses (PLD, DLD and ELD) were grouped under the umbrella term language disorders (LD) (Fig. 1). In this study, we divided the children with SSD into two diagnostic subgroups, motor speech disorder (MSD +) and language-oriented speech disorder (LD +). Depending on the SLP diagnoses they had received. MSD + diagnosis can be concomitant with oral motor developmental delay (ODD) and an LD diagnosis. LD + are children with no MSD diagnosis but can have concomitant ODD diagnoses.

### Assessment and procedure

The children’s assessments followed standardised procedures, and the OMC regular standardised assessment batteries were used. Three different regular standardised assessment batteries are used at OMC, depending on the referral question (detailed information is included in Appendix 1b). The assessment batteries support differential diagnosis by helping clinicians identify symptoms of speech motor delay, phonological disorders (e.g., impaired auditory discrimination), oral motor developmental delay, and structural oral dysfunctions. The child was seated on a chair with a table in front of the SLP, and the child could take a break if needed. If the child did not agree to cooperate, the procedure was ended. The assessments took between 30 min and 2 h, depending on the specific assessment battery. During the 2 h assessment, there was always at least one break with refreshments. All assessment results were registered in the children’s medical records by the assessing/examining SLP. After analysis of the results, a diagnosis was made, and comprehensive information was provided to the caregiver and to the clinician who made the referral.

### Chewing efficiency outcome measure

Chewing efficiency, with the Hue-Check chewing gum test, was introduced as part of the standard assessment batteries at the OMC when the research project started. This instrument was chosen because it can be evaluated clinically with a qualitative scale as well as by a quantitative method (Buser et al. 2018; Schimmel et al. 2015). All participants, including patients and controls, were assigned pseudonymised ID codes prior to assessment to ensure confidentiality and reduce risk of bias during data collection. The assessment was based on the colouring pattern of the 2-coloured chewing gum (one blue and one pink gum) Hue-Check after 20 chewing cycles. The SLP shows the two gums and instructs the child how to chew the gums together in 20 chewing bites and then spit them out. The gums were pressed together and placed on the tongue of the child with the blue gum facing down, and the SLP held the count of the chewing cycles. The gums were flattened to a wafer with a thickness of 1 mm using a 50 mm × 50 mm metal plate with a mild depression of 1 mm. For the analysis in this study, 101 chewing-gum specimens from the SSD group and 76 from the CG group were photographed with a smartphone from both sides by the responsible SLPs and dentists. The photographs were shared with the first author (HB), who documented and saved the samples on a hard drive. When all photos were gathered from the SSD and CG groups, they were sent to a calibrated expert with extensive experience assessing Hue-Check gum specimens. Chewing efficiency was evaluated using the optoelectronic analysis software View-Gum^©^, applied to Hue-Check gum specimens. The software summarises the images of the chewing gum specimen from both sides and transforms them into the HSI colour space. It then calculates the Variance of Hue (VOH), a colour dispersion metric reflecting how well the two colours have mixed. VOH is defined as 1 minus the length of the average hue vector. View-Gum^©^ presents the standard deviation between the two colour peaks as SDHue = √(VOH). This measure has a logarithmic association with the number of chewing cycles: a high VOH indicates poorly mixed colours due to inefficient chewing, whereas a low VOH reflects effective mastication and well-blended colours.

## Data analysis

To ensure that the groups were comparable in age and gender, descriptive statistics were calculated for all the studied groups (MSD +, LD +, and CG). The studied groups were divided into two age groups, 4–6 years of age and, 7–9 years of age. Due to suspected differences in chewing efficiency between the younger and older children as well as the different dental status e.g., from deciduous to mixed dentition. Descriptive statistics regarding mean, standard deviation, and confidence intervals, from the Hue-Check chewing gum test measured by SDHue were conducted for the two age groups for each studied group (MSD +, LD +, and CG). Descriptive statistics from the Hue-Check chewing gum test measured by SDHue were also conducted for children with a known NDD diagnosis and children with no NDD diagnosis separately. Shapiro–Wilk tests were conducted to examine whether the data for the two age groups (4–6 and 7–9 years of age) as well as for each studied group (MSD +, LD +, and CG) within each age group were normally distributed. A Mann–Whitney U test was used to examine if there were a difference in chewing efficiency between the two different age groups. To evaluate whether chewing efficiency differed between children with SSD in general and their typically developing (TD) peers, an independent samples t-test was used. This allowed for a direct comparison between the entire clinical group and the control group. To further explore differences within the SSD population, a one-way ANOVA was employed to detect any statistically significant differences in chewing efficiency across the three groups (MSD +, LD +, and CG). When the ANOVA indicated significant group effects, Tukey’s HSD post hoc test was carried out to identify specific between-group differences, while controlling for multiple comparisons.

All statistical analyses were performed using IBM SPSS Statistics, version 29.0.1.0 for Mac and all levels of significance were set at *α* < 0.05.

## Results

### Descriptive statistics

In the entire SSD group (SSD; *n* = 101), 59 (59%) children had two diagnoses, and 22 (22%) children had three diagnoses after assessment at OMC. Seventy-eight children (78%) children had received an MSD diagnosis, 41 children had ATYP, 18 children CAS, 11 children DYS, and eight children OA (some children received two or three concomitant MSD diagnoses). Among the MSD + subgroup (*n* = 78), 41 children had a concomitant LD diagnosis and 49 had received an ODD diagnosis (some children had both an LD and an ODD diagnoses). Additionally, 23 children with SSD had received a language disorder (LD) diagnosis without a co-occurring motor speech disorder (MSD). Among these, 15 children (15%) were diagnosed with phonological language disorder (PLD), 3 children (3%) with expressive language disorder (ELD), and 5 children (5%) with developmental language disorder (DLD). Within this LD-only group (LD +), 13 children (57%) were also diagnosed with oral motor developmental delay (ODD) following assessment at OMC. Detailed information of each specific diagnosis can be found in Fig. 1. Some children also had additional neurodevelopmental (NDD) diagnoses, including conditions within neuropsychiatry (NPD), as well as diagnoses related to genetics, cognition (e.g., intellectual disability, ID), and medical conditions. Seven children in the MSD + group age 4–6 years and four children age 7–9 years had received one or more NDD diagnosis and two children in the LD + group, one age 4–6 years and one age 7–9 years. No differences between MSD + and LD + groups were observed regarding age, gender, or NDD diagnosis (known NDD diagnosis yes or no). When comparing the entire SSD group with the CG group, a gender difference, but no age difference, was observed.

No differences in chewing efficiency were demonstrated between boys and girls [*t*(175) = 1.57,* p* = 0.12]. The studied groups (MSD +, LD +, and CG) were divided into two age groups, 4–6 years and 7–9 years. Normal distribution in all the studied groups was demonstrated using the Shapiro–Wilk test, except for the MSD + subgroup in the 7–9-year age range. In the LD + subgroup aged 7–9 years (*n* = 4), the result was considered inconclusive due to the small sample size. Based on these findings, the non-parametric Mann–Whitney U test was used. The analysis revealed a significant difference (*p*< 0.001) in chewing efficiency between the two age groups (4–6 and 7–9 years of age). Descriptive data of the studied groups and the results from the Hue-Check chewing gum test measured through SDHue are presented in Tables 1 and 2. Additional descriptive analyses were performed comparing children with and without a co-occurring NDD diagnosis in both the MSD + and LD + groups. The mean values, standard deviations, and confidence intervals were similar between children with and without NDD in the both the MSD + and the LD + groups, indicating comparable chewing efficiency regardless of NDD status. (Appendix 2).

**Table 2:** Descriptive results of chewing efficiency assessed with Hue-Check chewing gum test and measured through SDHue for age groups 4 to 6 and 7 to 9 years

Group	Age group	Mean (SD)	95% CI	*n*
CG	4–6 years	0.585 (0.184)	0.529–0.640	45
CG	7–9 years	0.327 (0.145)	0.273–0.380	31
SSD	4–6 years	0.610 (0.161)	0.572–0.648	71
SSD	7–9 years	0.550 (0.241)	0.460–0.640	30
MSD +	4–6 years	0.617 (0.161)	0.573–0.663	52
MSD +	7–9 years	0.580 (0.231)	0.487–0.673	26
LD +	4–6 years	0.586 (0.163)	0.508–0.665	19
LD +	7–9 years	0.353 (0.242)	−0.03–0.739	4

*CG* control group, *SSD* total speech sound disordered group, *MSD* + the subgroups motor speech disorders, *LD* + language-orientated speech disorders, Mean, *SD* standard deviation and *CI* confidence intervals are presented

### Analyses of chewing efficiency

**Fig. 2:**
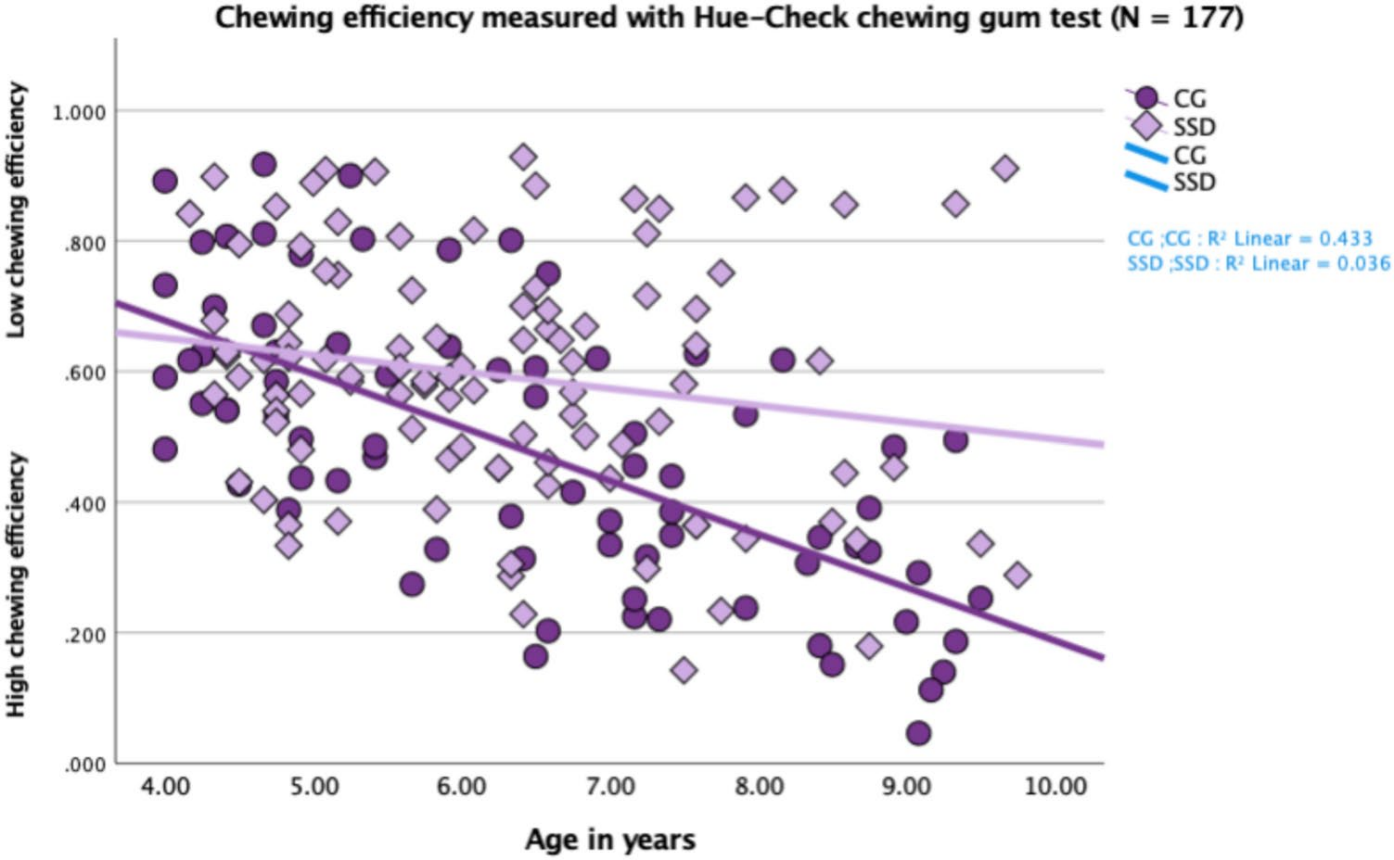
Scatterplot showing chewing efficiency measured through SDHue for the entire study group (SSD) compared to the control group (CG) age 4–9 years

**Fig. 3:**
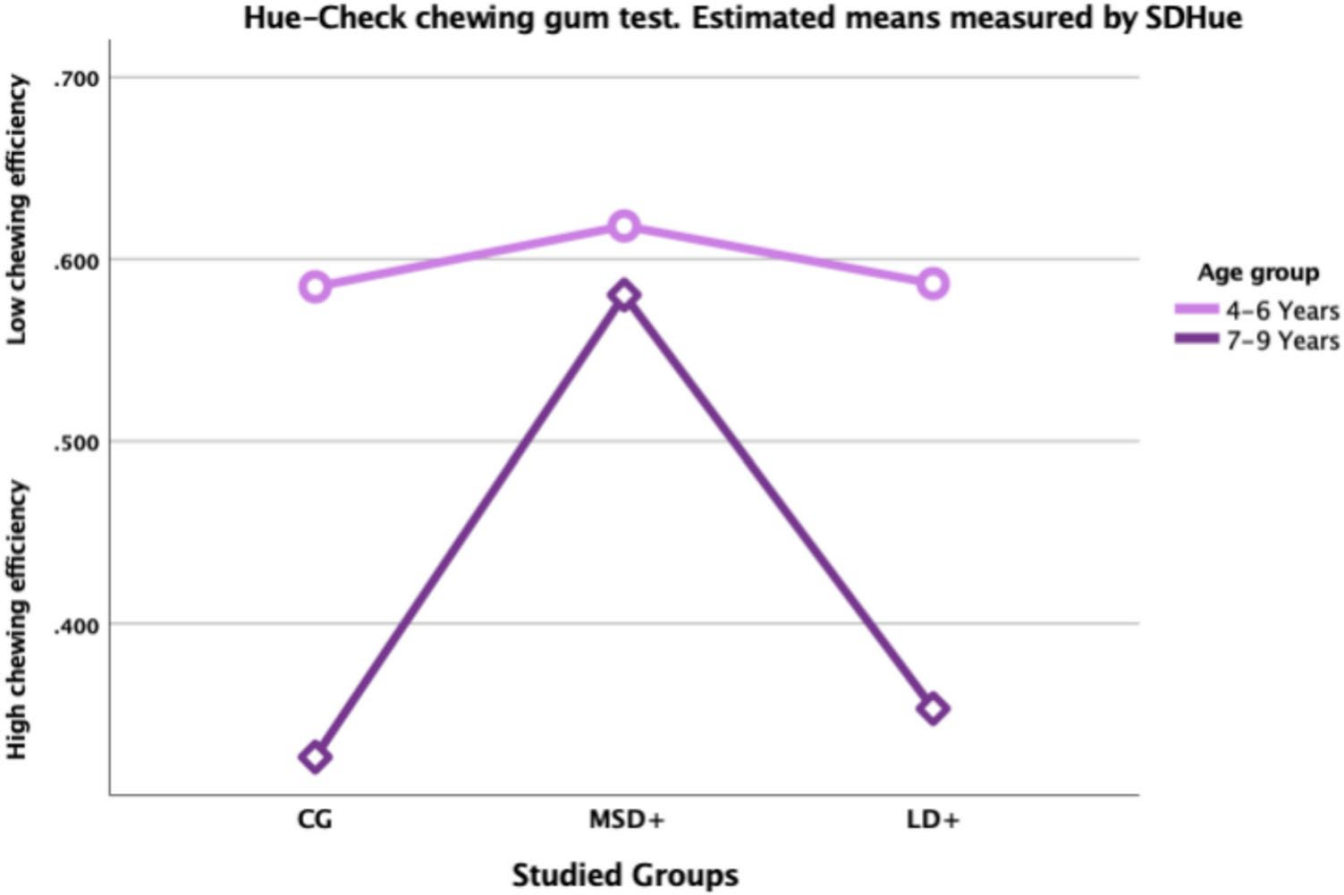
Profile plot showing the estimated mean of chewing efficiency measured through SDHue for the studied groups. Children diagnosed with motor speech disorders (MSD +) and language-oriented speech disorders (LD +) and typically developing children (CG). Age groups 4–6 and 7–9 years. For descriptives see Table 1

Figure 2 and Table 2 indicate a large variability in chewing efficiency in the entire cohort (*N* = 177). Furthermore, chewing efficiency seem to develop with age in TD children, (CG) (*r*^2^ = 0.433) but not for children in the SSD group (*r*^2^ = 0.036). Analyses conducted through an independent sample t-test showed that chewing efficiency was significantly lower in the SSD group (*t*(175) = 3.72, *p* < 0.001). To analyse differences in chewing efficiency between the groups (MSD +, LD +, and CG), a one-way ANOVA was conducted. The results indicated no significant difference in chewing efficiency between the groups for the 4- to 6-year-old children: *F*(2113) = 0.53, *p* = 0.589, *η*^2^ = 0.009. However, a significant difference was found for the children between 7 and 9 years of age: *F*(258) = 12.72, *p* < 0.001, *η*^2^ = 0.305. A post-hoc comparison using Tukey’s HSD test, demonstrated that children aged 7–9 years in the MSD + subgroup (*n* = 26) had significantly lower chewing efficiency compared to typically developing (TD) children (*n* = 31), (*p* < 0.001). Children in the LD + subgroup performed similarly to the TD group, though the result should be interpreted carefully due to the small sample size (*n* = 4) (Fig. 3). The comparison between MSD + and TD children showed a strong effect size (*η*^2^ = 0.305), indicating a substantial difference in chewing efficiency between these groups.

## Discussion

The present study investigated chewing efficiency in a clinical sample of 101 children with SSD and in 76 TD children 4–9 years of age. The children with SSDs showed less chewing efficiency in comparison to the TD children. However, this result may be due to that 7–9-year old children with MSD, demonstrated reduced chewing efficiency compared to the control group.

The children were referred to the OMC for evaluation of suspected speech motor disorders and were assessed using standardised procedures. The resulting diagnostic outcomes reflect the well-documented heterogeneity within this population, as most children in the study cohort received multiple diagnoses. Consistent with previous findings (e.g., Iuzzini-Seigel et al. 2022), a high prevalence of co-occurring language-related diagnoses were observed. Moreover, more than 50% of the children with speech sound disorders (SSD) also had a co-occurring diagnosis of oral motor developmental delay (ODD), a finding that has not been widely reported in the literature (Mogren et al. 2020). While ODD is not classified as a speech disorder, it may nevertheless influence the development and production of speech in ways that remain insufficiently understood (Kent 2015). Specifically, it may affect the planning and execution of speech-related motor patterns. From this perspective, symptoms associated with ODD could potentially impact what has been referred to as the"speech gesture."

Namasivayam et al. (2020) examined SSDs within an articulatory phonology framework, emphasising the speech motor milestones necessary for speech development. While they address the integration of phonological and motor processes, they do not explicitly consider the potential impact of delayed oral sensorimotor development. Notably, Namasivayam et al. (2020) acknowledge the importance of alternative explanatory models, an area where the current findings may offer valuable contributions. In the present sample, approximately 10% of the children had a diagnosed neurodevelopmental disorder (NDD), which is a known etiological factor in populations with motor speech disorders (MSD) (ASHA 2025). However, most children in this study had MSD of unknown origin. According to Shriberg et al., (2019), speech motor delay, referred to as atypical speech motor development (ATYP), may represent an idiopathic neurodevelopmental condition, for which ODD could plausibly be a contributing factor.

### Chewing efficiency

As a group, children with speech sound disorders (SSD) exhibited reduced chewing efficiency compared to typically developing (TD) children. Among children aged 7–9 years, those in the MSD + subgroup demonstrated significantly lower chewing efficiency than their TD peers, whereas children in the LD + subgroup performed at a level comparable to the TD group. However, this latter finding should be interpreted with caution due to the small sample size in the LD + subgroup. The strong effect size observed in the ANOVA supports the presence of a meaningful difference in chewing efficiency between children with and without motor speech difficulties. Chewing efficiency overall showed considerable variability, including among the TD children, which may be attributable to the wide age range of the study population (Almotairy et al. 2021; Alshammari et al. 2022; Kaya et al. 2017; Mogren et al. 2022a, b). While it may be expected that children with SSDs of known neurological or neuromuscular origin exhibit reduced chewing efficiency, this finding is more unexpected in cases of unknown aetiology. In the present study, children in the MSD + group demonstrated similar chewing efficiency regardless of whether a co-occurring NDD diagnosis was identified or not. Although chewing and swallowing difficulties are well documented in children with neuropathological conditions (Maynard et al. 2020), such impairments have not been previously described in children with MSD of unknown origin. Such results suggest that underlying sensorimotor or neurodevelopmental features associated with motor speech disorders (MSD) may contribute to, or interact with, chewing function (ASHA 2007, 2025; Kent 2015).

Chewing behaviour in typically developing (TD) children has been shown to develop along a continuum from early childhood through adolescence and into adulthood (Almotairy et al. 2021; Gisel 1991; Green et al. 1997). The present study identified clear age-related differences in chewing efficiency among TD children, with the younger group (ages 4–6) exhibiting lower efficiency compared to the older group (ages 7–9).

An important finding is that children in both the MSD + and LD + subgroups aged 4 to 6 years demonstrated chewing efficiency comparable to their TD peers. However, older children in the MSD + subgroup (ages 7–9) did not exhibit similar chewing efficiency relative to age-matched TD children. This observation supports the notion that persistent speech sound disorders (including motor speech disorders) beyond the age of 7 may be linked to missed developmental milestones, such as delayed sucking, gross motor delays, and speech development difficulties (Wren et al. 2016).

If chewing efficiency does not improve with age, it may negatively affect overall orofacial function and nutrition, potentially impacting quality of life (Chen & Engelen 2012; Mogren et al. 2022a, b). These findings underscore the clinical importance of evaluating chewing function in children with MSD, particularly in older age groups where developmental differences may be more pronounced.

Broadening the perspective to include both oral and general motor development, McAllister et al. (2013) observed that oral motor functions develop rapidly between the ages of 6 and 7. In terms of general motor development, children begin to accelerate their motor control abilities at around age 5, with continued progression until approximately 10 years of age, followed by a plateau between ages 12 and 18 (Gasser et al. 2010). The present study thus confirms that age-related development of oral motor control, as measured by chewing efficiency, is evident in TD children but not in those with MSD.

### Symptomatologic complexity

Speaking and chewing are governed by the same muscular system, although the extent of their interdependence remains a matter of debate (Bunton 2008; Kent 2015; Weismer 2006; Wilson et al. 2008). Mandibular control has been observed as early as nine months of age, developing in conjunction with sucking, chewing, and babbling (Steeve et al. 2008). Kent (2021) has suggested that intact oral motor milestones may support the development of vocalisation. The potential relationship between chewing and speech has been discussed particularly in the context of using non-speech oral motor tasks in intervention (Bunton 2008; Wilson et al. 2008). However, the limited empirical support for such tasks may have contributed to a relative underemphasis on oral motor development within speech research. In the present study, children with motor speech disorders (MSD) did not show age-related improvements in chewing efficiency, in contrast to their typically developing (TD) peers. This may suggest increased vulnerability to underlying oral motor deficits in children with speech motor difficulties (Mogren et al. 2022a, b). Recent attention has been directed toward the role of early-developing oral-sensory and motor functions in both feeding and speech (Kent 2021). These concepts are further elaborated in Kent’s Developmental Functional Modules (DFMs), which propose that early emerging sensorimotor functions provide fundamental support for later speech development. The reduced chewing efficiency observed in children with MSD may reflect a broader developmental trajectory and could be part of a larger symptom complex (cf. (Grillner 1982). Even if not directly implicated in speech production, inefficiencies in chewing remain clinically relevant and warrant attention in both assessment and intervention.

### Clinical implications

An overview of the children included in the present study confirms that diagnoses or developmental vulnerabilities rarely occur in isolation, and there is a risk that certain symptoms may be overlooked or insufficiently described. Oral symptoms are known to impact children’s emotional and social well-being (Abd-Elsabour et al. 2022; Chen and Engelen 2012) and should therefore be carefully considered in both the planning and implementation of treatment.

As such, clinical practice should incorporate assessment and intervention strategies that address both chewing efficiency and oral symptoms. Future research should adopt longitudinal designs and targeted interventions, with particular attention to identifying underlying causes, especially in children with motor speech disorders (MSD).

### Limitations

This study was conducted as part of routine clinical practice at the OMC, and participants consisted of a consecutive sample of all children aged 4–9 years who were referred to the clinic. To be eligible for inclusion, at least one caregiver had to be able to speak and read Swedish. Consequently, some children referred to the OMC were excluded from the study. Although interpreters are typically offered to caregivers who do not speak Swedish, this was not feasible in the context of the present study, which is part of a larger research project involving several questionnaires administered in Swedish. As a result, the sample may not fully represent the broader population served at the OMC.

Another limitation of this study is that the MSD group was not subdivided into specific subtypes. While our findings indicate that children with MSD generally exhibited the poorest chewing efficiency, not all children with SSDs showed such difficulties. Further subdivision of the MSD group was not feasible due to limited sample sizes. Additionally, the small sample size in the LD + subgroup aged 7–9 years limits the generalisability of findings related to this subgroup. Future research should aim to investigate chewing efficiency in larger and more demographically balanced samples to further investigate potential subgroup differences and underlying mechanisms within the SSD population.

There was also a limitation concerning the gender distribution within the study sample, which included more boys than girls, a distribution that was not exactly mirrored in the control group. Although no gender-related differences were observed in either the clinical group (SSD) or the control group (CG), this imbalance should be taken into account in future research.

Some limitations may also be associated with the chewing task. Although all clinicians involved were calibrated in the task procedure, minor variations in administration may have occurred. Furthermore, both speech- language pathologists (SLPs) and dentists reported that some younger children had difficulty following instructions and adhering to the procedure. While this may have influenced the quality of the chewing samples, no systematic group differences were observed in this regard.

## Conclusion

The present study demonstrates that children with motor speech disorders (MSD) exhibit reduced chewing efficiency compared to their typically developing (TD) peers. This impairment may represent part of a broader, yet poorly understood, symptom complex. Particularly given the notable prevalence of oral motor developmental delay (ODD) diagnoses among children with speech sound disorders (SSD). A comprehensive assessment of chewing and other oral functions in children with SSD is therefore essential and should be considered a standard component of the speech-language pathology (SLP) evaluation battery. These findings underscore the need for future research to explore symptom causality and to develop targeted interventions.

## Supplementary Information

Below is the link to the electronic supplementary material.

Supplementary file1 (DOCX 432 KB)

Supplementary file2 (DOCX 20 KB)

## Data Availability

No datasets were generated or analysed during the current study.
