# Supplementary material for: Chewing efficiency in children with motor speech disorders
Source: Eur Arch Paediatr Dent. 2025 Aug 20;27(1):75–85. doi: 10.1007/s40368-025-01095-6 (PMC12963083; doi:10.1007/s40368-025-01095-6)
Supplement: Supplementary file 1 — Supplementary file1 (DOCX 432 KB) [file 40368_2025_1095_MOESM1_ESM.docx]

**Appendix 1a**

References

- Hayden D. Differential diagnosis of motor speech dysfunction in children. Clinics in communication disorders. 1994;4(2):119-41.
- Caruso AJ, Strand EA. Clinical management of motor speech disorders in children: George Thieme Verlag; 1999.
- ASHA ASLHA. Childhood apraxia of speech [Technical report]. Rockville, MD; 2007.
- Thoonen G, Maassen B, Wit J, Gabreels F, Schreuder R. The integrated use of maximum performance tasks in differential diagnostic evaluations among children with motor speech disorders. Clinical linguistics & phonetics. 1996;10(4):311-36.
- Iuzzini-Seigel J, Allison KM, Stoeckel R. A tool for differential diagnosis of childhood apraxia of speech and dysarthria in children: A tutorial. Language, Speech, and Hearing Services in Schools. 2022;53(4):926-46.
- Murray E, Iuzzini-Seigel J, Maas E, Terband H, Ballard KJ. Differential Diagnosis of Childhood Apraxia of Speech Compared to Other Speech Sound Disorders: A Systematic Review. American Journal of Speech-Language Pathology. 2021;30(1):279-300.
- Namasivayam AK, Shin H, Nisenbaum R, Pukonen M, van Lieshout P. Predictors of Functional Communication Outcomes in Children With Idiopathic Motor Speech Disorders. Journal of Speech, Language, and Hearing Research. 2023:1-16.
- Kent RD. Nonspeech Oral Movements and Oral Motor Disorders: A Narrative Review. American Journal of Speech-Language Pathology. 2015;24(4):763-89.
- Kent RD. Handbook on Children's Speech: Development, Disorders, and Variations: Plural Publishing; 2023.
- McAllister A, Lundeborg I. Oral sensorimotor functions in typically developing children 3 to 8 years old; assessed by the Nordic orofacial test, NOT-S. J Med Speech Lang Pathol. 2013;21:51-9.
- Terband H, Maassen B, Guenther F, Brumberg J. Auditory–motor interactions in pediatric motor speech disorders: neurocomputational modeling of disordered development. Journal of communication disorders. 2014;47:17-33.

**Appendix 1b**
