# Supplementary material for: Chewing efficiency in children with motor speech disorders
Source: Eur Arch Paediatr Dent. 2025 Aug 20;27(1):75–85. doi: 10.1007/s40368-025-01095-6 (PMC12963083; doi:10.1007/s40368-025-01095-6)
Supplement: Supplementary file 2 — Supplementary file2 (DOCX 20 KB) [file 40368_2025_1095_MOESM2_ESM.docx]

Appendix 2

**Table 3.** *Descriptive statistics (Mean, Standard Deviation [SD] and 95% Confidence Intervals [CI]) of chewing efficiency assessed with the Hue-Check chewing gum test (SDHue) per age group. Presented in bold are the total subgroups of motor speech disorders (MSD+) and language-orientated speech disorders (LD+), in regular font broken down by with vs. without a known neurodevelopmental disorder (NDD)*

| Descriptive results of Hue-Check chewing gum test  analysed through SDHue | | | | |
| --- | --- | --- | --- | --- |
| Group | Age group | Mean (SD) | 95% CI | **n** |
| **MSD+** | **4-6 years** | **.617 (.161)** | **.573 - .663** | **52** |
| MSD+ with no NDD | 4-6 years | .630 (.156) | .583 - .677 | 45 |
| MSD+ with NDD | 4-6 years | .540 (.188) | .366 - .714 | 7 |
| **MSD+** | **7-9 years** | **.580 (.231)** | **.487 - .673** | **26** |
| MSD+ with no NDD | 7-9 years | .583 (.234) | .480 - .686 | 22 |
| MSD+ with NDD | 7-9 years | .566 (.259) | .154 - .977 | 4 |
| **LD+** | **4-6 years** | **.586 (.163)** | **.508 - .665** | **19** |
| LD+ with no NDD | 4-6 years | .583 (.238) | .509 - .674 | 18 |
| LD+ with NDD | 4-6 years | .501 ( - ) | - | 1 |
| **LD+** | **7-9 years** | **.353 (.242)** | **- .03 - .739** | **4** |
| LD+ with no NDD | 7-9 years | .239 (.010) | -. 008- .487 | 3 |
| LD+ with NDD | 7-9 years | .696 ( - ) | - | 1 |
